# Assessment of Dual Life Stage Antiplasmodial Activity of British Seaweeds

**DOI:** 10.3390/md11104019

**Published:** 2013-10-22

**Authors:** Jasmine Spavieri, Andrea Allmendinger, Marcel Kaiser, Maurice Ayamba Itoe, Gerald Blunden, Maria M. Mota, Deniz Tasdemir

**Affiliations:** 1 Department of Pharmaceutical and Biological Chemistry, School of Pharmacy, University of London, London WC1N 1AX, UK; E-Mails: Jasmine.Spavieri@ScienceMuseum.ac.uk (J.S.); andrea.allmendinger@gmx.de (A.A.); 2 Department of Medical Parasitology and Infection Biology, Swiss Tropical and Public Health Institute, Basel CH-4002, Switzerland; E-Mail: marcel.kaiser@unibas.ch; 3 University of Basel, Petersplatz 1, Basel CH-4003, Switzerland; 4 Instituto de Medicina Molecular, Universidade de Lisboa, Lisbon 1649-028, Portugal; E-Mails: mitoe@fm.ul.pt (M.A.I.); mmota@fm.ul.pt (M.M.M.); 5 School of Pharmacy and Biomedical Sciences, University of Portsmouth, Portsmouth PO1 2DT, UK; E-Mail: gs_blunden@dsl.pipex.com; 6 School of Chemistry, National University of Ireland, Galway, Ireland

**Keywords:** seaweed, marine alga, malaria, *Plasmodium*, malaria prophylaxis, fatty acid biosynthesis, liver stage, blood stage

## Abstract

Terrestrial plants have proven to be a prolific producer of clinically effective antimalarial drugs, but the antimalarial potential of seaweeds has been little explored. The main aim of this study was to assess the *in vitro* chemotherapeutical and prophylactic potential of the extracts of twenty-three seaweeds collected from the south coast of England against blood stage (BS) and liver stage (LS) *Plasmodium* parasites. The majority (14) of the extracts were active against BS of *P. falciparum*, with brown seaweeds *Cystoseira tamariscifolia*, *C. baccata* and the green seaweed *Ulva lactuca* being the most active (IC_50_s around 3 μg/mL). The extracts generally had high selectivity indices (>10). Eight seaweed extracts inhibited the growth of LS parasites of *P. berghei* without any obvious effect on the viability of the human hepatoma (Huh7) cells, and the highest potential was exerted by *U. lactuca* and red seaweeds *Ceramium virgatum* and *Halopitys incurvus* (IC_50_ values 14.9 to 28.8 μg/mL). The LS-active extracts inhibited one or more key enzymes of the malarial type-II fatty acid biosynthesis (FAS-II) pathway, a drug target specific for LS. Except for the red seaweed *Halopitys incurvus*, all LS-active extracts showed dual activity *versus* both malarial intracellular stage parasites. This is the first report of LS antiplasmodial activity and dual stage inhibitory potential of seaweeds.

## 1. Introduction

Malaria, a vector-borne disease caused by single-celled eukaryotic parasites from the genus *Plasmodium,* remains a major cause of global morbidity and mortality. According to World Malaria Report (2012), there were 219 million cases of malaria in 2010 and an estimated 660,000 deaths [1]. The disease is most prevalent in two continents, namely Africa and Asia, and the former is the most affected where approximately 90% of all malaria deaths occur [1]. Although the mortality rate has decreased in the last decade, the disease remains widespread in most endemic areas. The emergence of multiple drug resistance to front-line antimalarial drugs, including artemisinins, highlights the urgent need to identify new drugs from previously untapped sources.

The eradication or control of malaria has been more challenging than any other parasitic disease. One major reason for this is the complex life cycle of the parasite, involving two hosts (human and mosquito), three life stages [liver stage (LS), blood stage (BS) in human and mosquito stage (MS)] and both asexual and sexual multiplication. Infection in the human host begins when sporozoites are injected by the *Anopheles* mosquito during its blood meal. They migrate to the host liver where they multiply as LS (also called as exo-erythrocytic) forms during an asymptomatic incubation period of generally a week, before emerging in the blood stream and invading erythrocytes (BS). The constant and rapid replication cycle (1–2 days) of these merozoites results in several trillion parasites in erythrocytes [2]. In the case of *P. vivax* and *P. ovale*, some sporozoites develop cryptic forms, the so-called hypnozoites, which can lie silent in the liver for years before causing a relapse [3].

Antimalarial drug discovery has traditionally focused on targeting asexual BS. This has two reasons; first, it is the stage where the clinical manifestation of the disease emerges; second, the *in vitro* and *in vivo* test models of BS *P. falciparum* parasites are well established. Although an obligatory stage in the life cycle of the parasite whose blockage leads to a true causal prophylaxis of the disease [4], the non-symptomatic LS has remained underexploited due to technical and cost-related difficulties. Only recently developed medium and high throughput test systems allow screening of drug libraries against LS *Plasmodium* species [5]. While most antimalarial agents target BS, relatively few drugs inhibit the LS parasites. Primaquine, the only FDA licensed drug used clinically, acts against LS of all *Plasmodium* species and latent stage hypnozoites. However, its tendency to cause hemolytic anemia, short half-life and other drawbacks limit its use widely, complicating malaria control efforts [3,5]. As a result of long years of research, many biological targets have been identified and are being used for BS malaria drug discovery. However, the LS has been truly neglected in this sense and the biological target of primaquine is yet to be identified. Recently, a type II fatty acid biosynthesis (FAS-II) pathway, which takes place in a relict plastid like-organelle (the apicoplast) has been shown to be indispensable for the late stages of the LS parasites [6,7]. Disruption of the FAS-II system, which is different from the large associated human FAS-I system, prevents successful completion of the LS and the initiation of the BS infections, making FAS-II a valuable drug target for LS infections. The *de novo* FAS-II pathway was previously reported to operate in BS [8] to fuel significant research activity in this area. However, later transcriptome and proteome microarray studies showed that FAS-II genes are highly upregulated in LS [9]. Two subsequent studies [6,7] revealed the pathway to play a vital role only in the late phases of the LS schizogony. So it is timely to consider the potential of LS in current malaria intervention strategies and identify further LS targets. It is also essential to develop new drugs that target both mammalian developmental stages, providing true causal chemoprophylaxis and simultaneously curing BS malaria.

Most of the clinically used antimalarial drugs can be tracked to a natural product obtained from a terrestrial plant that has been used to treat malarial symptoms traditionally. For centuries, seaweeds (marine algae) have been consumed as traditional medicines to treat parasitic infections in many areas of the world, particularly in Japan and China [10]. Kainic acid was isolated as the active principle of the Japanese red alga *Digenea simplex* and used as an antihelminthic for a long time [11,12]. The diverse chemistry, ready access for wild harvesting, and cultivation possibility through mariculture makes seaweeds realistic sources for antiparasitic drug discovery, however their true potential has remained underexplored. Our group has been interested in the antiprotozoal potential of seaweeds from various parts of the world [13,14,15,16,17]. In the continuation of our research interest into this area, we herein report the *in vitro* activity of twenty-three British seaweeds against BS and LS malaria parasites. In an attempt to identify potential targets for LS activity, inhibitory activity of the seaweed extracts has also been determined against three key elongation enzymes of the *P. falciparum* FAS-II system (*Pf*FabI, *Pf*FabG and *Pf*FabZ), recombinantly prepared in our laboratory.

## 2. Results and Discussion

Twenty-three seaweeds (four green, eleven brown and eight red algae) were collected from the south coast of England. Table 1 shows their full taxonomical names, types, the exact collection sites and times. The seaweeds were extracted with CHCl_3_:MeOH mixtures, as described in the Material and Methods section, and used for biological activity assessments.

**Table 1:** British seaweeds used in the current study.

Seaweed Species	Code	Type	Family	Site of Collection	Time of Collection
*Cladophora rupestris* (L.) Kütz.	CR	Green	Cladophoraceae	Kimmeridge, Dorset	April 2007
*Codium fragile* (Sur.) Hariot ssp. *tomentosoides* (van Goor) Silva	CFsT	Green	Codiaceae	Kimmeridge, Dorset	April 2007
*Ulva intestinalis* L*.*	UI	Green	Ulvaceae	Kimmeridge, Dorset	April 2007
*Ulva lactuca* L.	UL	Green	Ulvaceae	Kimmeridge, Dorset	April 2007
*Ascophyllum nodosum* (L.) Le Jol.	AN	Brown	Fucaceae	Kimmeridge, Dorset	April 2007
*Fucus serratus* L.	FS	Brown	Fucaceae	Kimmeridge, Dorset	April 2007
*Fucus vesiculosus* L.	FV	Brown	Fucaceae	Kimmeridge, Dorset	April 2007
*Cystoseira baccata* (S.G.Gmel.) P.C.Silva	CB	Brown	Sargassaceae	Kimmeridge, Dorset	April 2007
*Cystoseira tamariscifolia* (Huds.) Papenf.	CT	Brown	Sargassaceae	Kimmeridge, Dorset	April 2007
*Sargassum muticum* (Yendo) Fensholt	SM	Brown	Sargassaceae	Kimmeridge, Dorset	April 2007
*Laminaria digitata* (Huds.) J.V. Lamour.	LD	Brown	Laminariaceae	Kimmeridge, Dorset	April 2007
*Saccorhiza polyschides* (Lightf.) Batt.	SP	Brown	Phyllariaceae	Kimmeridge, Dorset	April 2007
*Pylaiella littoralis* (L.) Kjellm.	PL	Brown	Acinetosporaceae	Emsworth, Hampshire	April 2007
*Leathesia difformis* (L.) Aresch.	LD	Brown	Chordariaceae	Kimmeridge, Dorset	August 2007
*Dictyota dichotoma* (Huds.) Lamour.	DD	Brown	Dictyotaceae	Hayling Island, Hampshire	July 2007
*Osmundea pinnatifida* (Huds.) Stackh.	OP	Red	Rhodomelaceae	Kimmeridge, Dorset	April 2007
*Calliblepharis jubata* (Good. et Woodw.) Kütz.	CJ	Red	Cystocloniaceae	Kimmeridge, Dorset	April 2007
*Ceramium virgatum* Roth	CV	Red	Ceramiaceae	Kimmeridge, Dorset	April 2007
*Claviclonium ovatum* (J.V.Lamour.) Kraft & Min-Thein	ClO	Red	Acrotylaceae	Kimmeridge, Dorset	April 2007
*Halopitys incurvus* (Huds.) Batt.	HI	Red	Rhodomelaceae	Kimmeridge, Dorset	April 2007
*Corallina officinalis* L.	CoO	Red	Corallinaceae	Seacombe, Dorset	April 2007
*Porphyra linearis* Grev	PoL	Red	Bangiaceae	Seacombe, Dorset	April 2007
*Halurus equisetifolius* (Lightf.) Kütz.	HE	Red	Wrangeliaceae	Kimmeridge, Dorset	August 2007

### 2.1. Blood Stage Antimalarial Activity and General Cytotoxicity of British Seaweeds

Due to the escalating problem of drug-resistance, the seaweed extracts were tested against BS parasites of the multi-drug resistant K1 strain of *P. falciparum*. A well-established radioactive incorporation assay utilizing ^3^H-Hypoxanthine was employed. [18]. As shown in Table 2, the extracts obtained from the brown algae *Cystoseira tamariscifolia* (CT) and *C. baccata* (CB), as well as the green alga *Ulva lactuca* (UL) exerted the highest erythrocytic stage antimalarial activity with IC_50_ values ranging between 3.3 and 3.8 μg/mL. The red seaweed *Corallina officinalis* (CoO) was also active (IC_50_ 8.6 μg/mL). The potency of the other ten active extracts, *i.e.*, green algae [*Cladophora rupestris* (CR), *Codium fragile* ssp. *tomentosoides* (CFsT) and *Ulva intestinalis* (UI), five brown algae (*Fucus serratus* (FS), *F. vesiculosus* (FV), *Sargassum muticum* (SM), *Laminaria digitata* (LD), *Saccorhiza polyschides* (SP) and two additional red algae (*Osmundea pinnatifida* (OP), *Ceramium virgatum* (CV)] varied from 11.8 to 18.2 μg/mL. The remaining nine extracts were completely inactive against BS malaria parasites at the highest test concentrations (50 μg/mL). The extracts were generally devoid of any cytotoxicity against mammalian L6 cells (IC_50_ values >90 μg/mL), a primary mammalian cell line derived from rat skeletal myoblasts [13,14,15]. Only the extracts of *C. tamariscifolia* (CT) and *C. officinalis* (CoO) showed low toxicity with IC_50_ values of 62.5 and 88.6 μg/mL, respectively (Table 2). In any case, the selectivity indices of algal extracts (SI: IC_50_ value obtained for mammalian cells divided by the IC_50_ value against the parasite) were over 10, including those of *C. tamariscifolia* (CT) and *C. officinalis* (CoO).

**Table 2:** *In vitro* inhibitory activity of seaweeds against *P. falciparum* blood stage (BS) and *P. berghei* liver stage (LS) infections, FAS-II target enzymes and mammalian L6 cells (IC_50_ values in μg/mL).

Seaweed Species	Type	BS	LS	*Pf*FabI	*Pf*FabG	*Pf*FabZ	L6 cells ^f^
*Cladophora rupestris*	Green	11.9	37.3	>50	>50	1.0	>90
*Codium fragile* ssp. *tomentosoides*		11.8	34.6	>50	>50	13	>90
*Ulva intestinalis*		18.2	>50	n.t	n.t	n.t	>90
*Ulva lactuca*		3.8	14.9	2.0	>50	7.0	>90
*Ascophyllum nodosum*	Brown	>50	>50	n.t	n.t	n.t	>90
*Fucus serratus*		17.6	>50	n.t	n.t	n.t	>90
*Fucus vesiculosus*		15.7	>50	n.t	n.t	n.t	>90
*Cystoseira baccata*		3.4	32.6	2.3	2.0	1.4	>90
*Cystoseira tamariscifolia*		3.3	49.4	37	2.8	1.3	62.5
*Sargassum muticum*		18.2	>50	n.t	n.t	n.t	>90
*Laminaria digitata*		17.6	>50	n.t	n.t	n.t	>90
*Saccorhiza polyschides*		16.1	>50	n.t	n.t	n.t	>90
*Pylaiella littoralis*		>50	>50	n.t	n.t	n.t	>90
*Leathesia difformis*		>50	>50	n.t	n.t	n.t	>90
*Dictyota dichotoma*		>50	>50	n.t	n.t	n.t	>90
*Osmundea pinnatifida*	Red	14.5	52.9	>50	>50	3.0	>90
*Calliblepharis jubata*		>50	>50	n.t	n.t	n.t	>90
*Ceramium virgatum*		13.6	26.4	30	13	4.2	>90
*Claviclonium ovatum*		>50	>50	>50	>50	2.1	>90
*Halopitys incurvus*		>50	28.8	2.4	15.9	2.9	>90
*Corallina officinalis* L.		8.6	>50	n.t	n.t	n.t	88.6
*Porphyra linearis*		>50	>50	n.t	n.t	n.t	>90
*Halurus equisetifolius*		>50	>50	n.t	n.t	n.t	>90
Positive control		0.056 ^a^	3.4 ^b^	0.014 ^c^	0.30 ^d^	0.03 ^d^	0.004 ^e,f^

Control drugs: ^a^ Chloroquine, ^b^ primaquine, ^c^ triclosan ^d^ (−)-epigallocatechin gallate, ^e^ podophyllotoxin, ^f^ from [15,16,17].

### 2.2. Liver Stage Activity of British Seaweeds

**Figure 1:**
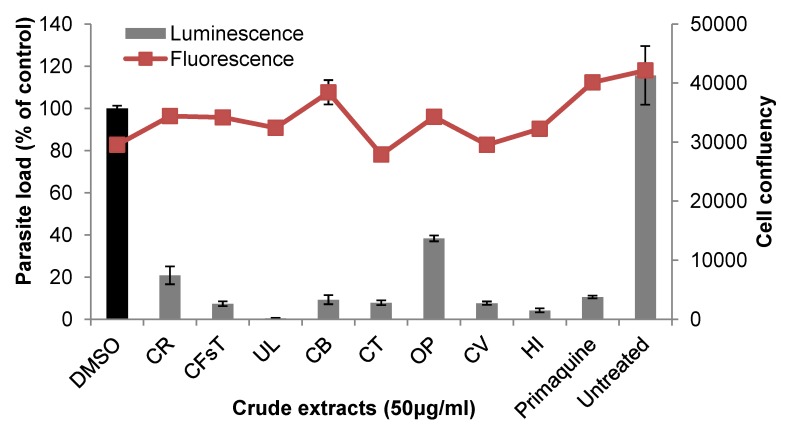
Inhibition of liver stage *P. berghei* infection by seaweed extracts at 50 μg/mL, determined by measurement of luciferase activity (bars, expressed as percentage of control) in PbGFP-Luc_con_-infected human hepatoma (Huh7) cells. Red line indicates the effect of the extracts on cell proliferation, assessed by fluorescence measurement of Huh7 cells at the time of analysis. Primaquine, positive control; DMSO-solvent-treated control. Error bars represent the standard deviations of three independent measurements. Abbreviations for the seaweed names can be found in Table 1.

**Figure 2:**
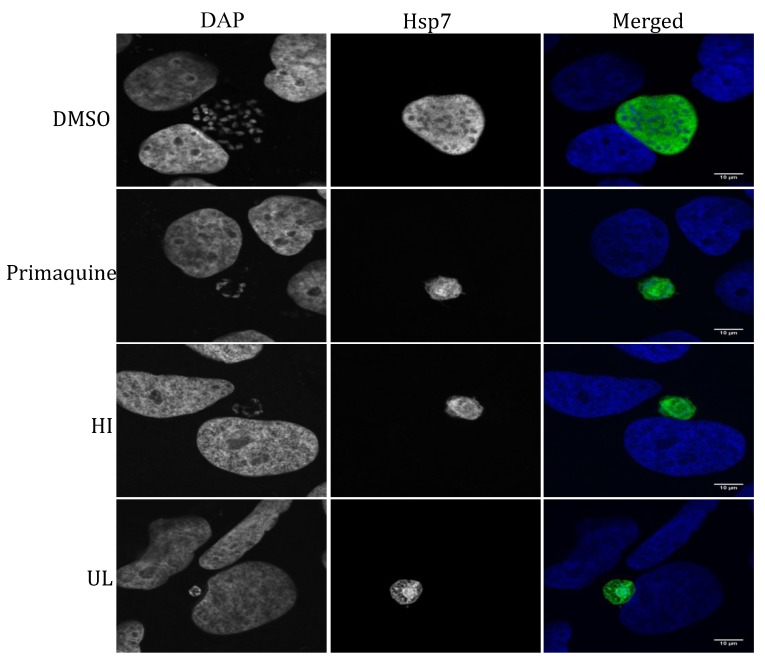
Immunostaining of *P. berghei* exo-erythrocytic forms (EEFs) at 44 h post infection (hpi). Huh7 cells were seeded on glass coverslips and infected with *P. berghei* ANKA sporozoites. At 3 hpi, infected cells were treated with control DMSO, 50 μg/mL of seaweed extracts, *Halopitys incurvus* (HI) and *Ulva lactuca* (UL), or primaquine (15 μM = 6.8 μg/mL). EEFs were immunostained with mouse anti-Hsp70 (Alexa 488, green). Nuclei were stained with DAPI (blue). Confocal images were acquired using a ZEISS LSM 510 confocal microscope with a X63 oil objective. Scale bar: 10 μM.

Next we tested the efficacy of the seaweed extracts on LS of the rodent malaria parasite *P. berghei* by using a transgenic *P. berghei* parasite expressing the bioluminescent reporter protein luciferase to visualize and quantify parasite development in Huh7 cells, a human hepatoma cell line. Eight seaweed extracts representing all three classes of marine macroalgae [green seaweeds *Cladophora rupestris* (CR), *Codium fragile* ssp. *tomentosoides* (CFsT), *Ulva lactuca* (UL); brown seaweeds *Cystoseira baccata* (CB), C. *tamariscifolia* (CT); red seaweeds *Osmundea pinnatifida* (OP), *Ceramium virgatum* (CV) and *Halopitys incurvus* (HI)], showed moderate activity against hepatic stage parasites with IC_50_ values in the range of 14.9–52.9 μg/mL (Table 2). Figure 1 displays the growth inhibitory activity of these extracts in human hepatoma (Huh7) cells, which were infected with luciferase-expressing *P. berghei* sporozoites and treated at 3 h post infection (hpi) with 50 μg/mL concentrations of seaweed extracts, DMSO and the positive control primaquine. Notably, none of the active extracts had any obvious effect on Huh7 cell viability, as assayed by fluorescence intensity measurements after incubation with an active plasma membrane labeling dye, Alamar Blue (red line, Figure 1). Intracellular localization of the LS parasites and the effect of the active extracts on the morphology and development of the LS parasites in Huh7 cells were studied by immunofluorescence analysis. Figure 2 shows representative confocal images of exo-erythrocytic form (EEFs) parasites in cells that were treated with two active extracts (HI and UL, 50 μg/mL), fixed, and labeled with anti-PbHSP70 (*P. berghei* heat shock protein 70, green). Both host cell and parasite nuclei were stained with Hoechst (blue). In accordance with the bioluminescence results, both extracts severely impaired the development of LS parasites, which was similar to that observed with 15 μM (=6.8 μg/mL) primaquine, the control drug.

### 2.3. Plasmodial FAS-II Enzyme Inhibitory Activity of British Seaweeds

The above-mentioned results on LS parasites encouraged us to search for potential drug targets of LS-active seaweed extract. We tested them, by using an established *in vitro* spectrophotometric assay [19], against three key elongation enzymes (*Pf*FabI, *Pf*FabG and *Pf*FabZ) of the *P. falciparum* type-II fatty acid biosynthesis (FAS-II) pathway. These are small (18.9–45.3 kDa) enzymes prepared recombinantly in our laboratory as described [19] and belong to classes of reductases (*Pf*FabI: Enoyl-ACP reductase, *Pf*FabG: beta-ketoacyl-ACP reductase) and dehydratases (*Pf*FabZ: Beta-hydroxyacyl-ACP dehydratase). The rationale for the selection of the enzymes was based on the fact that the *Pf*FabI and *Pf*FabZ enzymes are vital for pre-erythrocytic (liver) stage infection [6,7]. The *Pf*FabG was also included, as its inhibition has been shown to cause anti-LS activity [9]. As depicted in Table 2, half of the extracts (brown: *Cystoseira baccata* (CB), *C. tamariscifolia* (CT), red: *Ceramium virgatum* (CV), *Halopitys incurvus* (HI), inhibited all three target enzymes (IC_50_ values 1.3–37 μg/mL). The green algae *Cladophora rupestris* (CR), *Codium fragile* ssp. *tomentosoides* (CFsT), and the red algae *Osmundea pinnatifida* (OP) and *Claviclonium ovatum* (ClO) inhibited only the dehydratase *Pf*FabZ at low μg/mL doses (IC_50_ values 1.0–13 μg/mL). The crude extract of *U. lactuca* (UL) was the only one that inhibited both *Pf*FabI and *Pf*FabZ with IC_50_ values of 2 and 7 μg/mL.

Considering the serious concerns of drug resistance to BS antimalarials, and difficult-to-treat chronic liver stages, plus the need for a true causal prophylaxis, it is obvious that ideal malaria therapy should target both intracellular mammalian stages of the parasites. This study provided a simultaneous profiling of 23 seaweeds collected from British waters for both LS and BS activity in two different *Plasmodium* species in standardized conditions. The aim of this profiling was (i) to assess stage selectivity of the seaweed extracts, (ii) to discover new resources for the discovery of malaria prophylactic natural products that target LS, and (iii) most importantly, to find new natural resources that can possess dual stage activity against both stage parasites. More than half of the seaweed extracts studied here revealed good to moderate level of BS activity against human malaria parasite *P. falciparum*, whereas eight extracts impaired the LS cultures of rodent malaria parasite *P. berghei*. Eight extracts failed to kill either LS or BS parasites *in vitro*. The green alga *U. intestinalis* (UI),brown algae *Sargassum muticum* (SM), both *Fucus* species (FS, FV), *Laminaria digitata* (LD), *Saccorhiza polyschides* (SP), and red alga *Corallina officinalis* (CoO), inhibited BS parasites only, whereas *Halopitys incurvus* (HI) selectively targeted the LS parasites. Importantly, except for the latter extract, all extracts that displayed LS growth arrest also killed BS parasites, indicating that they are inhibitors of both intracellular stages of *Plasmodium*. Although the potency against LS is generally 2- to 3-fold lower than that of BS activity, it is still significant that, for the very first time, LS activity is being detected in seaweed extracts. To the best of our knowledge, this is the first report of hepatic stage antiplasmodial activity and dual activity of any seaweed in the literature. The extracts with anti-LS activity inhibited one or more LS target enzymes of the FAS-II. Whether FAS-II could be considered as the ultimate target of the LS-active extracts is unclear at this stage, and needs to be further validated by metabolic studies to confirm the inhibition of parasitic fatty acid biosynthesis by the seaweed extracts. Importantly, the extracts do not show any significant toxicity on Huh7 cells or L6 cells, indicating selectivity, which is important for drug development. This is also the first study describing the BS antimalarial activity of British seaweeds.

A detailed survey indicates that only a few recent screening studies have investigated the BS inhibitory activity of the seaweed extracts originating from Turkey, France, Egypt and India [13,14,20,21,22,23,24]. Some of the macroalgae tested in this study have been included in the previous studies, but the results are only partly in agreement with ours. The discrepancy might stem from the use of different strains of *Plasmodium* species and different methods. Two studies that have used the similar experimental conditions are worthy of mention. The first screening study performed on seaweeds collected from the Normandy coast (France) showed ethyl acetate extracts of red and brown seaweeds to be highly active against the multi-drug resistant K1 strain of *P. falciparum* [20]. The green algal extracts, including that of *U. lactuca* (UL) that appeared to be one of the most active extracts in the present work, were not active. In contrast, *Dictyota dichotoma* (DD), which was completely inactive in our study showed considerable activity (IC_50_ 8.8 μg/mL). On the other hand, *U. lactuca* (UL) and *C. barbata* (CB) collected from Turkish waters revealed much lower activity against the same K1 strain of *P. falciparum* (IC_50_ 18.3 and 48.8 μg/mL, respectively) [13,15]. The discrepancy is likely to originate from the use of different solvents for extraction. Another major factor is the environment from which the seaweeds originate, as the environmental pressures shape the chemical composition, hence the biological activity of the marine organisms. This study reports the LS and/or BS activity of a few seaweed genera, e.g., *Osmundea*, *Halopitys*, *Fucus and Saccorhiza* for the first time.

Limited studies have reported BS antiplasmodial activity of both primary and secondary metabolites obtained from marine macroalgae. Most of the antiplasmodial seaweed secondary metabolites belong to the class of terpenes, mainly isolated from red and brown algae. The examples include halogenated terpenes [25], brominated meroterpenes [26,27,28], tocopherol derivatives [27] and carotenoid-like compounds [29]. Interestingly, complex primary metabolites, such as sulfated polysaccharides were also shown to inhibit both drug-sensitive and drug-resistant strains of *P. falciparum* and the adhesion of parasitized erythrocytes to the human glycoprotein CD36 [30]. Fucoidan, another sulfated polysaccharide from the Korean brown seaweed *Undaria pinnatifida* inhibits the invasion of *Plasmodium* merozoites into erythrocytes *in vitro* and shows *in vivo* activity [31]. Large polysaccharides, e.g., fucoidan, also hinder the binding of sporozoites to the surface of hepatocytes and thus invasion into these cells [32]. Hence, it is intriguing to study both the LS and BS activity of our extracts to identify both primary and/or secondary metabolites responsible for the initial activity, and identify their mechanism(s) of actions.

The malaria parasite *Plasmodium* has two distinct intracellular growth stages inside the human host. Despite taking place in two distinct cell types (LS in hepatocytes, BS in erythrocytes); having an orders-of-magnitude difference in parasite replication (LS ten thousands, BS trillions); the number of cycles (LS one cycle, BS multiple cycles) and length (LS generally 7 days, can be up to 14 days depending on *Plasmodium* species; whereas BS 1–3 days), the LS and BS have many common features. In both stages, the parasite forms a parasitophorous vacuole (PV) in which it grows and replicates safely [33]. Many metabolic pathways are presumably similar [9]. A recent screening identified several compounds with anti-LS activity, many of which inhibit similar metabolic pathways to BS targets, such as dihydrofolate reductase or cyctochrome bc1 complex [5]. Replication of the malaria parasites in the liver or in erythrocytes requires vast amounts of fatty acids (FA). *Plasmodium* was previously thought to acquire FAs by scavenging from the human host. However, the parasite was shown to be capable of type II fatty acid biosynthesis (FAS II) [34], whose enzymes are targeted to the apicoplast, a relict, non-photosynthetic plastid of red algal origin [35]. Interestingly, the pathway is only essential in the LS, mainly during the transition stage from LS to BS to initiate red blood cell infection [6,7]. Triclosan was previously reported to inhibit BS infections, *in vitro* and *in vivo*, through inhibition of *Pf*FabI, a rate-limiting FAS-II enzyme. Triclosan is a potent *Pf*FabI inhibitor, but its BS activity in rodent models was shown to be minimal [36]. It inhibits the late LS of *Plasmodium* [37]. Currently, it is not fully understood why the FAS-II pathway is necessary for the late LS, but it is probably due to requirement of a sheer amount of membrane biogenesis for the formation of very large numbers of merozoites in BS [7]. In this study we show that red algae, as well as other types of marine algae, are capable of inhibiting at least one algal-like *Pf*FAS-II enzyme, and probably thereby impair the LS parasite growth. A multiple enzyme inhibition capacity is an advantage, as it will require much longer time for the parasite to introduce mutations, if any, hence bear a low/slow risk of resistance development against the drug. The apicoplast harbors other plant-bacterial type pathways, such as non-mevalonate isoprene, lipoic acid and heme biosyntheses [38]. It would be intriguing to investigate whether the complex algae extracts inhibit these pathways as well. Potential drug targets for BS activity warrant further studies.

It is now widely accepted that malaria control or eradication can only be achieved through a multidisciplinary approach, which requires research and investment in multiple developmental life stages of the parasite and evaluation of hitherto untapped sources, such as marine organisms. The results obtained in this study can be useful in several ways. First, it can further fuel investigations in finding seaweed metabolites than can cure BS malaria infections, in line with the traditional malaria drug discovery efforts. Secondly, seaweed extract(s) that selectively inhibit the LS only (e.g., HI) can be studied to identify natural products that provide true causal chemoprophylaxis. Such a strategy has certain advantages, e.g., low risk of resistance, due to low consumption rates of the compound and low numbers of parasites that exist during liver stages. Finally, seaweed extracts can be studied to reveal new metabolites with dual stage efficacy for both preventing and curing malaria infections. Compounds with multiple stage activity have the potential to be very beneficial for controlling the spread of disease in malaria endemic areas and for preventing disease in travelers to malarious regions.

Malaria drug discovery from natural sources, particularly from marine organisms faces many obstacles, such as the minute yield of the active principles and the complexity of the chemical structures for synthesis. The use of seaweeds in drug discovery is more advantageous; as the supply issue is less problematic in comparison to that observed with other marine organisms (e.g., invertebrates). Furthermore, it is nowadays possible to cultivate seaweeds by using aqua- or mariculture to obtain larger amounts of the biomass and the bioactive compounds of interest. Hence, seaweeds merit intensive studies as source of highly efficacious, new and affordable lead molecules for prevention or therapy of malarial infections.

## 3. Experimental Section

### 3.1. Algal Material

The algal material was collected from different locations along the south coast of England in 2007. Table 1 includes details on their origin and collection times. The samples were initially identified by one of the authors (GB) and verified by Dr. W.F. Farnham (Department of Biological Sciences, University of Portsmouth, Portsmouth, Hampshire, UK). Voucher samples of the algae are kept in the Herbarium of the Hampshire County Council Museums Service (available under Bi 2000.16) and at the School of Chemistry, National University of Ireland, Galway, Ireland (under voucher numbers GB07).

### 3.2. Preparation of the Extracts

Freshly collected algae (*ca.* 10–20 g wet weight) were soaked in *i*-PrOH (100 mL) and transported to the laboratory. Algal material was homogenized by a household blender and filtered. The residue was extracted overnight with CHCl_3_:MeOH mixtures (3:1 and 1:1, respectively) under continuous stirring at room temperature (rt) and combined with the *i*-PrOH extract obtained above. The combined crude extract was dissolved in 10 mL EtOAc:MeOH (1:1) mixture and centrifuged. The supernatant was evaporated to dryness in vacuum at 30 °C and kept at −20 °C until used in the biological assays.

### 3.3. Blood Stage Antiplasmodial Activity

*In vitro* activity against erythrocytic stages of *P. falciparum* was determined by a modified [^3^*H*]-hypoxanthine incorporation assay [18], using the chloroquine- and pyrimethamine-resistant K1 strain and the standard drug chloroquine. Briefly, parasite cultures incubated in RPMI 1640 medium with 5% Albumax (without hypoxanthine) were exposed to serial drug dilutions in microtiter plates. After 48 h of incubation at 37 °C in a reduced oxygen atmosphere, 0.5 μCi ^3^H-hypoxanthine was added to each well. Cultures were incubated for a further 24 h before they were harvested onto glass-fiber filters and washed with distilled water. The radioactivity was counted using a Betaplate^TM^ liquid scintillation counter (Wallac, Zurich, Switzerland). The results were recorded as counts per minute (CPM) per well at each drug concentration and expressed as percentage of the untreated controls. IC_50_ values were calculated from graphically plotted dose-response curves by linear regression [39] using Microsoft Excel. The reported IC_50_ values are the means of at least two separate experiments.

### 3.4. Liver Stage Antiplasmodial Activity

#### 3.4.1. Culturing and Infection of Huh7 Cells with *P. berghei* ANKA Sporozoites

Human hepatoma cells (Huh7, 1–4 × 10^4^) were seeded per well of a 96 well-plate or on glass coverslips in a 24 well-plate, respectively, and incubated overnight at 37 °C in a 5% CO_2_ incubator. Luciferase-expressing transgenic *P. berghei* sporozoites that were dissected from salivary glands of infected *Anopheles stephensi* mosquitoes were re-suspended in an appropriate volume of RPMI 1640 medium supplemented with 10% Fetal Bovine Serum (FBS), 1% penicillin-streptomycin, 10mM HEPES, 1% glutamine, 1% non-essential amino acids, and 0.02% Fungizone. Ten to thirty thousand sporozoites were added to each well of 96- or 24-well plates (for luciferase or microscopy assays, respectively), followed by centrifugation at 3000 rpm for 5 min and incubation at 37 °C in a 5% CO_2_ incubator for the duration of the experiment.

#### 3.4.2. *In Vitro* Testing of Liver Stage Antiplasmodial Activity of Seaweed Crude Extracts

Three hours after infection, the seaweed extracts were diluted in complete culture medium to a final concentration of 50 μg/mL and added to infected cells for over 40 h with medium/crude extracts changes 24 h after infection. Dimethysulfoxide (DMSO, Sigma-Aldrich, Munich, Germany), at a final concentration of 1.15% was used as a negative control. Primaquine biphosphate (Sigma Aldrich, Munich, Germany) was the positive control. Antiplasmodial activity of seaweed crude extracts was assayed according to ref. [40]. Briefly, at the end of treatments, cells were rinsed twice with PBS and further incubated in 75 μL of 1× reconstituted firefly luciferase lysis buffer per well, with agitation for 15–20 min at 400 rpm at rt. The plate was centrifuged at 1000 rpm for 5 min in order to pellet cellular debris. Thirty μL of lysate from each well was transferred into a well in a white 96 well plate (Corning Incorporated, Corning, NY, USA), followed by the addition of 50 μL of 0.2 mg/mL of d-luciferin. The plate was agitated for 4 s and luminescence intensity was measured in a microplate plate reader (Infinite M200, Tecan, Group Ltd., Männedorf, Switzerland) for 100 ms at excitation and emission wavelengths of 530 nm and 590 nm respectively. The level of luciferase activity in drug-treated samples was expressed as a percentage of control. All measurements were run in triplicates.

#### 3.4.3. Immunofluorescence Detection of *P. berghei* Parasites in Extract- or Control-Treated Huh7 Cells

For intracellular localization of *P. berghei* parasites in Huh7 cells by immunofluorescence, *P. berghei*-infected cells on glass cover slips that had been treated with 50 µg/mL of each seaweed extract (or DMSO (1.1%), as a negative control) were washed briefly with PBS and fixed immediately with 4% paraformaldehyde (PFA) for 14 min at rt. The cells were washed thrice with PBS for 5 min on a shaker and then incubated in 0.1 M glycine for 10 min to quench cellular autofluorescence. Cellular membranes were permeabilized by incubation in 0.1% saponin for 20 min at rt, followed by three washes with PBS. The cells were further incubated in blocking solution, 1% bovine serum albumin (BSA) with 0.05% saponin, for 1 h at rt. *P. berghei* parasites were stained with a parasite specific anti-Hsp70 (2E6) antibody at a 1:300 dilution for 1 h at rt, followed by three washes with blocking solution and further incubation in a 1:400 dilution of an anti-mouse Alexa 488 secondary antibody. A further three washes were carried out with blocking solution in the presence of a 1:1000 dilution DAPI. Cover slips were mounted on microscope slides with fluoremount. Confocal images were acquired using a laser scanning confocal microscope, LSM 510 META, with the following parameters: excitation with 405 nm, Band Pass (BP): 420 nm–480 nm, excitation with 488 nm, BP: 505 nm–530 nm. Images were processed with freely available Image J software.

#### 3.4.4. Cell Viability

The effect of crude extract (or DMSO as a negative control) on the viability of Huh7 cells was assayed by the Alamar Blue fluorescence method [41] prior to the lysis of infected Huh7 cells. Briefly, cells were incubated with a 1/20 dilution of Alamar Blue in complete RPMI 1640 medium for about 90–120 min and the fluorescence intensity was measured at 595 nm in a microplate reader (InfiniteM200, Tecan, Group Ltd., Männedorf, Switzerland).

### 3.5. Plasmodial FAS-II Enzyme Inhibition Assays

Plasmodial FAS-II enzyme inhibitory assays were carried out (in duplicate) as previously described [19]. Briefly, all measurements were performed in 20 mM HEPES, pH 7.4, and 150 mM NaCl in a total volume of 1 mL and *Pf*FabZ activity was measured at 263 nm for 2 min in the presence of 25 μM crotonoyl-CoA and 1 μg of enzyme. Reference compounds were triclosan (*Pf*FabI) and (−)-epigallocatechin gallate (*Pf*FabG, *Pf*FabZ).

### 3.6. General Cytotoxicity against Mammalian Cells

Assays were performed in 96-well microtiter plates, each well containing 100 μL of RPMI 1640 medium supplemented with 1% l-glutamine (200 mM) and 10% FBS, and 4 × 10^4^ L6 cells. Serial drug dilutions of seven 3-fold dilution steps. were prepared. After 72 h of incubation the plates were inspected under an inverted microscope to assure growth of the controls and sterile conditions. Ten μl of a resazurin solution (12.5 mg resazurin in 100 mL water) was added to each well and the plates incubated for another 2 h. The plates were read with a Spectramax Gemini XS microplate fluorometer using an excitation wavelength of 536 nm and an emission wavelength of 588 nm. Data were analyzed using the microplate reader software Softmax Pro. Podophyllotoxin was the control drug.

## 4. Conclusions

Due to the limited arsenal of antimalarials, it has become necessary to find novel compounds to target multiple life stages of the malaria parasite. The current study paves the way for high throughput screening of seaweeds, and is hoped to encourage research for exploitation of seaweeds in discovery of natural products acting as dual inhibitors of two key developmental stages. Our future studies will include chemical profiling of the active extracts by LC-MS-based dereplication techniques and purification/structure elucidation of the active metabolites with dual activity by employing activity-guided isolation procedure. Mechanism of action studies will also be performed to identify common and/or specific drug targets of these compounds in both stages.
